# Therapeutic Effects of Propolis Supplementation on the Inflammatory State, Oxidative Stress, and Uremic Toxins in dialysis Patients: A Systematic Review

**DOI:** 10.1002/fsn3.72309

**Published:** 2026-09-08

**Authors:** Mahshid Mardani, Alireza Ostadrahimi, Azizeh Farshbaf‐Khalili

**Affiliations:** ^1^ Faculty of Nutrition Tabriz University of Medical Sciences Tabriz Iran; ^2^ Nutrition Research Center Tabriz University of Medical Sciences Tabriz Iran; ^3^ Physical Medicine and Rehabilitation Research Center, Aging Research Institute Tabriz University of Medical Science Tabriz Iran

**Keywords:** bee glue, dialysis, hemodialysis, peritoneal dialysis, Propolis, systematic review

## Abstract

Chronic renal failure (CRF) is a global health issue, particularly affecting the elderly. The end stage of CRF requires dialysis that it often results in complications such as inflammation, oxidative stress, infections, and various kidney issues. Propolis, a natural bee product rich in antioxidants, may help mitigate these risks for dialysis patients. To search for related articles, we utilized the Cochrane Library, EMBASE, ClinicalTrials.gov, Google Scholar, MEDLINE via PubMed, Web of Science, Scopus, and SID databases without any time limitation, up to May 2025. We included all clinical trials, with or without randomization, and congress abstracts that studied the efficacy of Propolis supplementation in patients receiving dialysis. The quality and risk of bias of the included articles were evaluated in accordance with the guidelines outlined in the Cochrane Collaboration Handbook. Six trials (*n* = 225 patients/48.4 ± 13.0 years) were included, with Propolis dosages of 200–400 mg/day for 2–6 months. Across studies, inflammatory markers decreased by approximately 8%–42%, including significant reductions in TNF‐α (−22.6%, *p* < 0.001), IL‐8 (−41.9%, *p* = 0.009), IL‐17 (−20.0%, *p* = 0.039), and IFN‐γ (−8.3%, *p* = 0.005) after 8 weeks of Brazilian green Propolis supplementation. Additional trials reported significant reductions in TNF‐α levels (*p* = 0.009 and *p* = 0.020), while CRP and IL‐6 generally remained unchanged (*p* > 0.05). Oxidative stress improved, with oxLDL levels decreasing by approximately 12.9% (946–824 pg/mL, *p* = 0.020), although MDA showed no significant changes. Topical Propolis suppressed catheter‐site infections (0% vs. 6.7%–10% in controls), although this was not statistically significant. Adverse effects were not mentioned in any study. There were small changes in uremic toxins and gut microbiota. Current evidence suggests that Propolis supplementation may improve inflammatory and oxidative stress biomarkers in patients undergoing dialysis and appears to have an acceptable safety profile. However, because substantial methodological heterogeneity precluded a quantitative meta‐analysis, these findings should be interpreted with caution. Larger, well‐designed randomized controlled trials are required before definitive clinical recommendations can be made.

## Graphical abstract

This systematic review of 6 clinical trials (*n* = 225) demonstrates that Propolis supplementation (200–400 mg/day for 2–6 months) significantly reduces key inflammatory cytokines TNF‐α, IL‐8, IL‐17, IFN‐γ, and oxidative stress (oxLDL) in dialysis patients. While Propolis demonstrates an acceptable safety profile and potential for infection risk reduction, methodological heterogeneity necessitates larger, well‐designed RCTs before establishing definitive clinical practice guidelines.

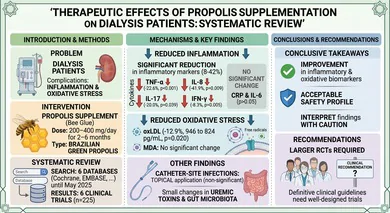

## 1 Introduction

Chronic renal failure (CRF) is one of the most significant global public health concerns, particularly for the elderly. With a global prevalence exceeding 10%, CRF affects over 800 million individuals, leading to substantial healthcare costs for nations (Kovesdy 2022). This alarming trend is evident in Iran, where the number of CRF cases increased from 97,300 in 1990 to 315,500 in 2019, highlighting the growing epidemic of kidney disease (Shahbazi et al. 2023). Its progressive nature results from irreversible renal damage, and disease severity is categorized into five stages based on glomerular filtration rate (GFR). The final stage (GFR < 15 mL/min/1.73 m^2^), known as end‐stage renal disease (ESRD), requires immediate treatment as accumulated toxins and fluid overload become life‐threatening (Tattersall et al. 2011). Dialysis is the primary treatment modality for ESRD, acting as an artificial kidney to remove waste products and maintain homeostasis (Hakim and Lazarus 1995; Shahbazi et al. 2023; Tattersall et al. 2011). Both major dialysis methods, hemodialysis using extracorporeal filtration devices and peritoneal dialysis utilizing the peritoneal membrane, offer distinct advantages but share similar drawbacks (Hakim and Lazarus 1995). While dialysis is life‐sustaining, its repetitive nature paradoxically leads to serious complications. The treatment generates oxidative stress through various mechanisms, including immune activation at the dialysis membrane interface, where complement proteins and IgG trigger granulocyte recruitment (Nagane et al. 2013). These activated immune cells generate reactive oxygen species (ROS) while simultaneously depleting endogenous antioxidants like superoxide dismutase (SOD) and trace elements such as copper and zinc (Macunluoglu et al. 2016; Nagane et al. 2013). Additionally, the dialysis process promotes chronic inflammation characterized by elevated acute‐phase reactants like C‐reactive protein, ferritin, and interleukin‐6, along with persistent activation of the complement system (Eustace et al. 2004; Jofré et al. 2006; Weaver et al. 2017). This inflammatory‐oxidative cascade places patients at risk for numerous comorbidities, including anemia, coagulopathies, recurrent infections, electrolyte disturbances, and notably, accelerated cardiovascular disease (Araujo et al. 2012; Han et al. 2017; Stenvinkel et al. 1999). The growing use of dialysis for increasingly mild renal insufficiency highlights the urgent need for comprehensive education for both patients and practitioners regarding its associated risks (Araujo et al. 2012). Current pharmacologic therapies for dialysis complications remain inadequate, creating a demand for adjunctive therapies that are safer and more effective (Silveira et al. 2019). Propolis, a natural resinous compound also known as “bee glue,” has emerged as a promising candidate due to its unique biochemical profile. Produced by honeybees from plant exudates, wax, and salivary enzymes, Propolis serves as a protective mechanism for the hive and contains over 300 bioactive constituents. Phenolic acids and flavonoids contribute to its strong antioxidant activity, while caffeic acid phenethyl ester (CAPE) exhibits significant anti‐inflammatory properties through cytokine modulation (Batista et al. 2012; Bazmandegan et al. 2017; Braakhuis 2019; Cornara et al. 2017; Mirzoeva and Calder 1996) (Table 1). Experimental evidence supports the biphasic activity of CAPE in suppressing pro‐inflammatory mediators (TNF‐α, IL‐1β) and stimulating the production of anti‐inflammatory cytokines (IL‐10, IL‐4), as well as inhibiting inflammatory cell infiltration (Abdel‐Latif et al. 2005; Rajoo et al. 2014; Moura et al. 2011). Although occasional allergic reactions may occur, Propolis demonstrates an excellent safety profile with minimal systemic toxicity, while also protecting the vascular endothelium and reducing oxidative damage (Silveira et al. 2021, 2019). Despite these pharmacological benefits, there is no comprehensive evaluation of Propolis supplementation in dialysis patients. Therefore, this systematic review aims to critically assess the impact of Propolis on three key parameters in dialysis patients: inflammatory markers, oxidative stress markers, and uremic toxin accumulation. By synthesizing available evidence, the present study will address a crucial knowledge gap and potentially identify a new therapeutic option for managing dialysis‐related complications.

**TABLE 1:** Bioactive compounds of Propolis.

Chemical classifications	Compounds	Bioactivities
Phenols	5‐Pentadecyl Resorcinol 3,4‐Dihydroxyphenylethanol 1,2‐Dihydroxybenzene	Antifungal activity (Oliveira et al. 2022) Antidermatophytic Action (Romagnoli et al. 2016) Anti‐oxidant and neuroprotective effect (Cheng et al. 2019) Antimicrobial activity, Cytoprotective activity (Harbatsevich et al. 2017; Krishna et al. 1992)
Phenolic acids	Gallic acid Cinnamic acid Caffeic acid 3,4‐Dimethyl‐caffeic acid Ferulic acid	Antioxidant and antibacterial activities (Kerdsomboon et al. 2021; Wu et al. 2022) Neurological Activity, lowering blood glucose, and Anti‐inflammatory activity (Lan et al. 2017) Enhancement of atherosclerosis, Alzheimer's disease, and Anticancer (Singh et al. 2018)
Flavonoids	Apigenin Pinobanksin Kaempferol Pinocembrin Epigallocatechin Genistein Pinobanksin‐5‐methylether acetate Kaempferol‐4′‐methyl ether Naringenin Quercetin Isorhamnetin 3,5,7,4′‐Tetrahydroxy‐3′‐methoxyflavylium	Anticancer, Antioxidant, and Anti‐inflammatory Effects (Salehi et al. 2019) Cardiovascular disease (Dabeek and Marra 2019), Neuroprotective against cerebral ischemic injury, and antimicrobial activities (Rasul et al. 2013) Anti‐Alzheimer activities (Lambert et al. 2006) Antiallergic activities (Liu et al. 2022)
Flavanones, flavones, flavonols, and derivatives	Rutin Pinobanksin‐3‐*O*‐acetate Chrysin Gallocatechol Myricetin Pinobanksin‐3‐(E)‐caffeate Catechol Catechin Esculetin Techtochrysin Myricetin‐3,7,3′‐trimethyl ether 4,2′,4′‐Trihydroxy‐2‐methoxychalcone 5,7‐Dihydroxyflavanone 5‐Hydroxy‐7‐methoxyflavanone 5,7‐Dihydroxyflavone 3,5,7‐Trihydroxyflavone	Antioxidant, antimicrobial, and Anticancer activities (Bangar et al. 2022; Kim and Lim 2019) Hepatoprotective, attenuate psoriasis‐like skin lesions (Li et al. 2020; Song et al. 2020) Anti‐inflammatory, Neuroprotective effect, Wound healing, Enhancing the absorption of functional foods and their antioxidant properties (dos Santos 2020; Puertas‐Bartolomé et al. 2021; Taheri et al. 2020) Inhibit skin hyperpigmentation, treat yellow plague, malaria, and diarrhea (Pillaiyar et al. 2017) Antihyperuricemic and renal protective effects (Jia et al. 2023)
Alkylphenols	2‐Hydroxyl‐6‐(14′Z‐nonadecenyl) benzoic acid 3‐Undecyl phenol 3‐Tetradecylphenol 3‐Heptadecylphenol 3‐(12′*Z*‐Heptadecenyl)‐phenol	Antimicrobial activity (Schultz et al. 2006) Potential antioxidant properties (Ruangviryachai et al. 2000) Antiproliferative, antileishmanial, and Anti‐inflammatory effects (Kardar et al. 2014)
Terpenoid	Germacrene D Sandaracopimaric acid	Anti‐bacterial efficacy, and Anti‐inflammatory activity (Ren et al. 2010)
Phenylpropanoids	(E)‐5‐Hydroxy‐1,7‐diphenylhept‐1‐ene‐3‐acetate (E)‐3‐Hydroxy‐1,7‐diphenylhept‐1‐ene‐5‐acetate	Antibacterial, and antioxidant effects (Proteggente et al. 2002)
Vitamins	Vitamin C Vitamin E	Antioxidant activities (Proteggente et al. 2002)
Amino acids	Pyroglutamic acid	Antifungal, and antibacterial activities (Gang et al. 2018)
Aldehyde	Cyclohex‐1‐en‐1‐ carboxaldehyde	Antivirus effects (Tania et al. 2023)

## 2 Materials and Methods

This systematic review was conducted in accordance with the guidelines of the Cochrane Handbook for Systematic Reviews of Interventions (Higgins et al. 2024) and the Preferred Reporting Items for Systematic Reviews and Meta‐Analyses (Shamseer et al. 2015). All interventional studies, including randomized controlled trials (RCTs) as well as quasi‐ and semi‐experimental studies, that investigated the effects of Propolis supplementation on health outcomes in patients undergoing dialysis were included. This study was approved by the Research Ethics Committee (IR.TBZMED.REC.1405.110) and was also registered on the PROSPERO website for systematic reviews (ID: CRD420251054868).

### 2.1 Inclusion Criteria

In this review, the PICOS framework “participants, intervention, comparison, outcomes, and study design” was used to define the inclusion criteria. The participants consisted of patients (both over and under 18) undergoing dialysis (hemodialysis or peritoneal dialysis). The intervention involved using Propolis extract supplements in various forms or dosages. The comparison groups included placebo, other established supplements, or no intervention. Outcomes covered all health‐related effects of supplementation, including physical, psychological, and metabolic outcomes. Eligible study designs included randomized controlled trials (RCTs), quasi‐experimental studies, and semi‐experimental studies (Table 2).

**TABLE 2:** PICOS criteria for inclusion and exclusion of studies.

Parameter determined criteria for the present study	
Participants	Patients undergoing dialysis (hemodialysis/peritoneal dialysis)
Intervention	Propolis extract without restrictions regarding dose and duration
Comparator	Placebo, Routine treatment
Outcomes	Changes in inflammation state, oxidative stress state, and renal function
Study design	Clinical trials, both double‐armed and single‐armed.

Propolis was administered at any stage of dialysis treatment, including both hemodialysis and peritoneal dialysis, as well as during the follow‐up period of patients receiving dialysis‐related medical care. All relevant studies involving dialysis patients at any stage were considered eligible. This review included Propolis supplementation in any form, such as capsules or ointments, administered at any dosage and for any duration, provided it was used alone and not combined with other nutrients. No age limitations were applied.

### 2.2 Exclusion Criteria

Studies were excluded if they were review articles, animal experiments, or cellular and molecular investigations. In addition, study protocols, observational designs (including cross‐sectional, cohort, and case–control studies), as well as case reports and case series, were not considered eligible. Non‐original publications such as letters to the editor and editorials were also excluded from the review. Studies including patients with CRF, ARF, or ESRD who were not under dialysis treatment or were under dialysis but did not receive any Propolis (different natural remedies or Propolis mixed with other substances) were excluded.

### 2.3 Types of Outcomes

The primary outcome was to determine the therapeutic effects (inflammatory state, oxidative stress level, and uremic toxicity level changes) of supplemental administration of Propolis (bee glue) in dialysis patients. The secondary outcome was to determine the safety of Propolis supplementation and possible side effects.

### 2.4 Search Strategy and Study Selection

A comprehensive systematic search of the literature was conducted across multiple electronic databases, including the Cochrane Central Register of Controlled Trials, Web of Science, MEDLINE (PubMed), Scopus, EMBASE, Google Scholar, and ClinicalTrials.gov, in addition to the Scientific Information Database (SID), IranDoc, and IranMedex for Persian‐language studies. The search was performed up to May 2025 using Medical Subject Headings (MeSH) terms combined with Boolean operators (see Appendix S1).

Keywords, either used individually or in combination, were applied to identify articles related to dialysis and Propolis supplementation. The search included both published and ahead‐of‐print studies without restrictions on time, location, or language. In addition, the reference lists of all included articles were manually screened to identify any studies not captured in the database search. Conference proceedings were also examined for relevant evidence. EndNote X8 software (Thomas Reuters) was used to organize and manage the retrieved records generated through the search strategy.

Two independent reviewers (M.M. and A.F‐.K.) separately evaluated the eligibility of studies on the effects of Propolis supplementation in dialysis patients by screening the titles and abstracts of all identified records. Any disagreements arising during the screening process were resolved through discussion with a third reviewer (A.O.). The same procedure was followed during the full‐text review stage.

### 2.5 Data Extraction

Data from all eligible studies were independently extracted by three reviewers (M.M., A.F‐.K., and A.O.). The relevant information was recorded in a standardized electronic form developed for this review, which included the author and year of publication, country, study design, study objectives, assessed outcomes, sample size, intervention and control groups, duration of follow‐up, main findings, and any reported adverse effects (Table 3).

**TABLE 3:** The characteristics of the included studies investigating the effects of Propolis supplementation on patients undergoing dialysis.

ID	Authors (year)	Country	Study design	Type of disease	Study objectives	Intervention group (IG), control group (CG), (*n* = number), duration	Participants	Results data (e.g., *p* values)	Adverse events & safety
1	Moghiseh et al. (2022)	Iran	Double blind randomized clinical trial	CKD	To evaluate the effect of Propolis on catheter exit site infection (ESI) and peritonitis in peritoneal dialysis (PD) patients.	IG: 0.9% normal saline, 10% Propolis extract ointment.(*n* = 30) CG: 0.9% normal saline+2% mupirocin ointment rub.(*n* = 30) for 6 months of duration and follow‐up once every 2 weeks.	Ninety patients undergoing peritoneal dialysis (both male and female), aged between 18 and 60 years, who receive dialysis at least twice a day, have had at least 3 months since their last treatment. The start of PD	10% of patients in the placebo group and 6.7% in the control group developed catheter exit site infections, while no patients in the intervention group developed this infection (*p* = 0.469). Similarly, 6.7% of the placebo and control groups developed peritonitis, but no patients in the intervention group contracted peritonitis (*p* = 0.997). No significant differences in catheter exit site infections and peritonitis incidence were observed among the three groups.	No adverse events were observed.
2	Duarte Silveira et al. (2022)	Brazil	A prospective, open‐label, proof‐of‐concept, single‐center clinical study	ESRD with CKD	The primary endpoint was the assessment of inflammatory parameters using the interleukin and hs‐CRP panel. The secondary outcome was the safety assessment of EPP‐AF through an adverse effect questionnaire and laboratory tests.	IG: 200 mg/day Propolis extract (Propomax/EPP‐AF) Capsules, one 100 mg capsule twice a day (*n* = 37). No control Group. For 4 weeks of duration, followed by 4 weeks of control (without the drug). There was a wash‐out week between periods.	Thirty‐seven patients with a mean age of 58.6 ± 15 years underwent the same hemodialysis treatment during the study, which included the following: classic hemodialysis mode; three times a week; blood flow ranging from 300 to 350 mL/min; an average dialysate flow of 500 mL/min; capillary FX 100 classix or FX 80 classix, using a Fresenius 4008S machine (Fresenius Medical Care AG, Bad Homburg, Germany); and an adjusted time sufficient for a single pool KT/V of greater than or equal to 1.2.	Patients exhibited an exacerbated inflammatory state at baseline. During EPP AF use, there was a significant reduction in IFN‐c (*p* = 0.005), IL‐13 (*p* = 0.042), IL‐17 (*p* = 0.039), IL‐1ra (*p* = 0.008), IL‐8 (*p* = 0.009), and TNF‐α (*p* < 0.001) levels compared to baseline, the Hs‐CRP levels showed a trend toward improvement. The heatmap revealed a pattern of pronounced pro‐ inflammatory status at baseline, particularly in patients with primary glomerulopathies, and a clear reduction in this pattern during the use of EPP‐AF. There was a tendency to maintain this reduction after discontinuation of EPP‐AF.	No adverse events were observed.
3	Baptista et al. (2023)	Brazil	Double blind randomized clinical trial	CKD	To evaluate the effects of the Propolis supplement. Evaluation of inflammatory markers in patients with CKD on PD.	IG: 400 mg/day capsules containing concentrated and standardized dry EPP‐AF green Propolis extract,4 capsules of 100 mg. (*n* = 12). CG: 400 mg/day containing magnesium stearate, silicon dioxide, and microcrystalline cellulose,4 capsules of 100 mg, ingested twice a day, 2 capsules with lunch and dinner.(*n* = 10) For 2 months of duration.	Twenty‐two patients were randomized; however, three were lost to follow‐up, leaving 19 patients analyzed at the end: 10 in the Propolis group and 9 in the placebo group. Patients with CKD on PD for more than 3 months, aged between 20 and 80 years, and following an individualized dietary prescription of 25 to 35 kcal/kg/day and 1.0 to 1.2 g/kg/day of protein, as recommended by the NKFKDOQI, 2020, were included in the study.	The plasma levels of TNF‐α reduced significantly (*p* = 0.02), and the expression of Nrf2 showed a trend toward an increase (*p* = 0.07) after Propolis supplementation; however, no statistical change in MDA, IL‐6, and CRP plasma levels was observed.	No adverse events were observed.
4	Chermut et al. (2023)	Brazil	Double blind randomized clinical trial	CKD	To evaluate the effects of Propolis supplementation on inflammatory markers in patients with CKD on HD.	IG: 400 mg/day (4 capsules of 100 mg/day) containing concentrated and standardized dry EPP‐AF green Propolis extract.(*n* = 21) CG: 400 mg/day (4 capsules of 100 mg/day) containing microcrystalline cellulose, magnesium stearate, and colloidal silicon dioxide.(*n* = 20) For 2 months of duration.	Forty‐one patients completed the follow‐up. They had chronic kidney disease (CKD) and were on hemodialysis (HD) for at least 6 months. The patients were aged between 18 and 75 years, utilized arteriovenous fistula (AVF) as vascular access, and received individualized dietary prescriptions, which included an adequate energy supply of 25–35 Kcal/ideal Kg/day and protein intake ranging from 1.0 to 1.2 g/ideal Kg/day as recommended by NKF‐KDOQI, 2020. The HD sessions lasted from 3 to 4.5 h and occurred three times a week, with a dialysate flow of 500 mL/min and a blood flow greater than 250 mL/min.	The obtained data revealed that the intervention with Propolis significantly reduced the serum levels of tumor necrosis factor α (TNFα) (*p* = 0.009) and tended to reduce the levels of macrophage inflammatory protein‐1β (MIP‐1β) (*p* = 0.07). There were no significant differences noted in the placebo group.	No adverse events were observed.
5	Fonseca, Alvarenga, et al. (2024)	Brazil	Double blind randomized clinical trial	CKD	This study aimed to evaluate the effects of green Propolis extract on inflammatory and oxidative stress markers in patients with CKD on HD.	IG: 400 mg/day (consisting of 4 capsules, each containing 100 mg/day) of concentrated and standardized dry EPP‐AF green Propolis extract. (*n* = 9). CG: 400 mg/day (consisting of 4 capsules, each containing 100 mg/day) of microcrystalline cellulose, magnesium stearate, and colloidal silicon dioxide. (*n* = 15). For 2 months.	Twenty‐four patients concluded the study: 9 patients in the Propolis group (50.0 ± 7.0 years, 4 men, 22.6 ± 6.9 kg/m^2^) and 15 in the placebo group (46.0 ± 13.5 years, 5 men, 26.1 ± 5.8 kg/m^2^).	After supplementation, oxLDL plasma levels decreased in the Propolis group, going from 946 (766–1212) pg/mL to 824 (700–1011) pg/mL (*p* = 0.02). In the placebo group, there was no statistically significant difference before and after the intervention, with levels changing from 1073 (805–1331) pg/mL to 825 (695–1078) pg/mL (*p* = 0.13).	No adverse events were observed.
6	Fonseca, Ribeiro, et al. (2024)	Brazil	Double blind randomized clinical trial	CKD	To evaluate the effects of Propolis on the gut microbiota profile and uremic toxin plasma levels in HD patients.	IG: 400 mg/day Propolis extract (EPP‐AF) Capsules. (*n* = 21) CG: 400 mg/day microcrystalline cellulose, magnesium stearate, and colloidal silicon dioxide capsules.(*n* = 20) For 2 months of duration.	Forty‐one patients with an arteriovenous fistula (AVF) aged between 18 and 75 years old, with CKD on HD for at least 6 months and with dietary prescription (adequate energy supplying 25–30 Kcal/ideal Kg/day and protein from 1.0 to 1.2 g/ideal kg/day according to the recommendation by NKF‐KDOQI 2020) were included in the study.	There was a positive correlation between IAA and TNF‐α (*r* = 0.53, *p* = 0.01), IL‐2 (*r* = 0.66, *p* = 0.002), and between pCS and IL‐7 (*r* = 0.46, *p* = 0.04) at baseline. No significant changes were noted in the levels of uremic toxins after the intervention. Although not statistically significant, microbial evenness and observed richness increased following the Propolis intervention. Counts of Fusobacteria species showed a positive correlation with IS, while counts of Firmicutes, Lentisphaerae, and Proteobacteria phyla exhibited a negative correlation with IS. Two months of Propolis supplementation did not lower the plasma levels of uremic toxins (IAA, IS, and p‐CS) or alter the fecal microbiota.	No adverse events were observed.

*Note:* *p* < 0.05 was considered statistically significant.

Abbreviations: CG, control group; CKD, chronic kidney disease; CRP, C‐Reactive Protein; ESRD, end stage renal disease; HD, Hemodialysis; Hs‐CRP, high sensitive C‐reactive protein; IAA, Indole‐3‐acetic acid; IG, intervention group; IL, Interleukin; IL‐1ra, Interleukin‐1 Receptor Antagonist; INF‐c, interferon‐c; IS, Indoxyl Sulfate; MDA, Methylenedioxyamphetamine; Nrf2, Nuclear Factor Erythroid 2‐related Factor 2; oxLDL, Oxidized‐Low‐Density Lipoprotein; p‐CS, p‐Cresyl Sulfate; PD, Peritoneal Dialysis; TNF‐α, Tumor Necrosis Factor alpha.

### 2.6 Assessment of Methodological Quality

The risk of bias and overall quality of the included studies were evaluated by two independent reviewers (M.M. and A.F‐.K.) in accordance with the Cochrane Collaboration Handbook guidelines. The assessment included random sequence generation (selection bias), allocation concealment (selection bias), blinding of participants and personnel (performance bias), blinding of outcome assessment (detection bias), incomplete outcome data (attrition bias), and selective reporting (reporting bias). Any disagreements were resolved through consultation with a third reviewer (A.O.). The risk of bias results were illustrated using Review Manager 5.3 (RevMan; The Cochrane Collaboration, Oxford, UK) software.

### 2.7 Data Synthesis

Data were synthesized using a qualitative approach and presented as a systematic review due to insufficient data for quantitative pooling. One study was a single‐arm interventional trial, while the remaining five studies reported outcomes that were not sufficiently comparable to allow for meta‐analysis.

## 3 Results

### 3.1 Literature Search and Study Selection

**FIGURE 1:**
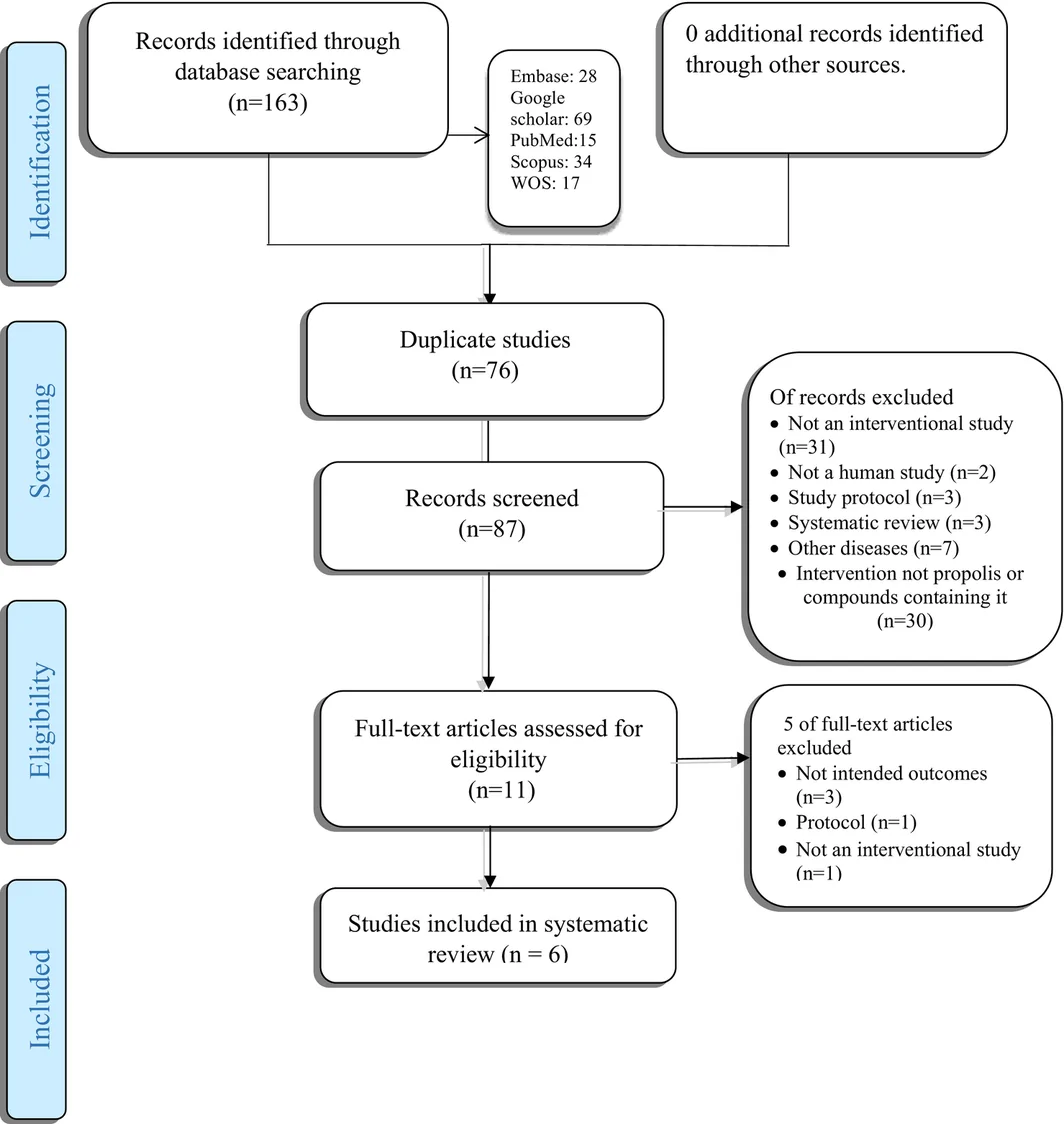
Study's flow diagram for the search and selection process of articles considered in this review.

The details of the search process, study selection, and reasons for exclusion are shown in Figure 1. A total of 163 records were identified across multiple databases. After removing 76 duplicates, the remaining studies were screened for eligibility. Of the 87 articles assessed, 31 were excluded for not being interventional studies, 2 for not involving human participants, 3 for being systematic reviews, 3 for study protocols, 7 for focusing on other renal replacement therapies, and 30 for evaluating supplements other than Propolis or using Propolis in combination with other substances. Ultimately, 11 studies were selected for full‐text review, of which 6 met the eligibility criteria and were included in the final systematic review.

### 3.2 Description of the Studies

The selected trials in this systematic review were published from 2022 to 2024. Of the six articles reviewed, five contained full‐text in English, and one had only an English abstract (Fonseca, Alvarenga, et al. 2024; Fonseca, Ribeiro, et al. 2024). Among the included articles, five were randomized controlled trials (RCTs), while one was a single‐arm uncontrolled before–after trial (Duarte Silveira et al. 2022). Additionally, five studies were conducted in Brazil, and one in Iran (Moghiseh et al. 2022). The total number of participants across all studies was 225. All participants were undergoing different types of dialysis, either hemodialysis or peritoneal dialysis. Propolis extracts were used as the sole supplement in all studies. In five studies, Propolis was administered orally, typically in capsule form, and in one, it was applied as an ointment (Moghiseh et al. 2022). In most studies, the dosage and duration of the interventions were comparable. Daily Propolis intake varied between 200 and 400 mg, while the intervention duration ranged from 2 to 6 months. Comprehensive details of all included articles are presented in Table 3.

### 3.3 Risk of Bias in the Included Studies

**FIGURE 2:**
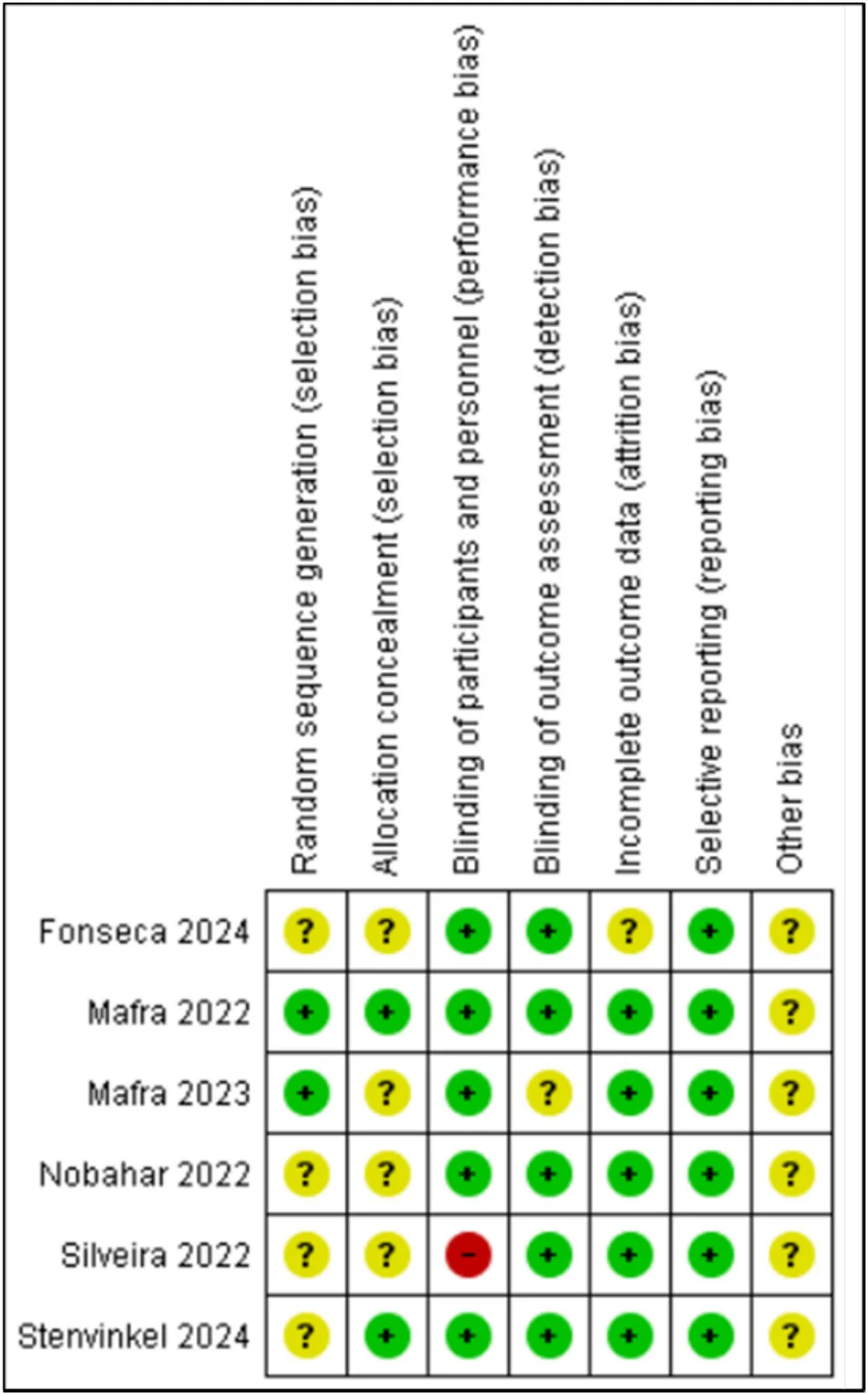
Risk of bias summary: review authors' judgments about each risk of bias item for each included study.

**FIGURE 3:**
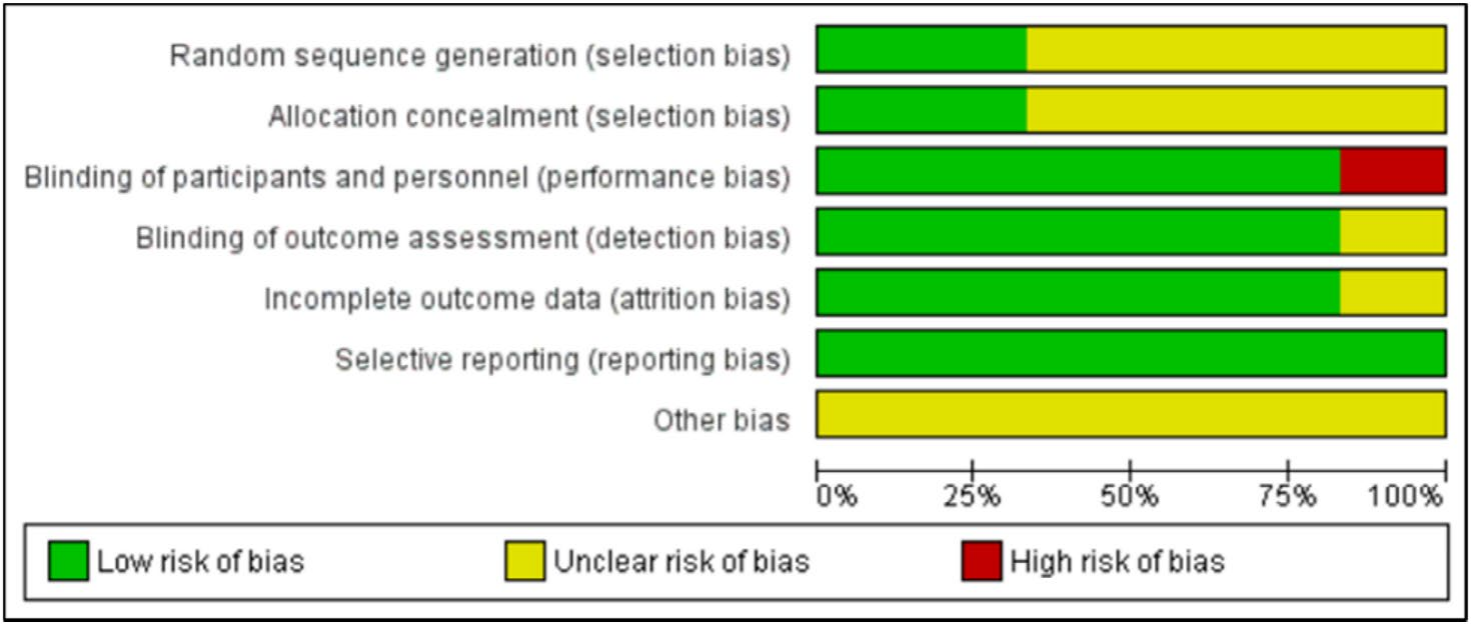
Risk of bias graph: review authors' judgments about each risk of bias item presented as percentages across all included studies.

The methodological quality of each included study, based on the authors' assessment of risk of bias, is presented in Figures 2 and 3. Overall, most of the included studies were considered to be of high methodological quality. The selection bias was low risk between 25% and 50%, and performance bias, detection bias, and attrition bias were all over 57% low risk. Reporting bias was 100% low risk.

### 3.4 Effectiveness of the Interventions

Moghiseh et al. (2022) conducted a 6‐month study comparing Propolis, mupirocin, and saline for preventing infection in patients on PD for at least 3 months. Patients applied the assigned treatment on alternate days at the PD exit site. The Propolis group showed a 0% incidence of exit‐site infections (ESI) and peritonitis, compared to mupirocin, which had a 6.7% (2/30) ESI incidence, and saline, which had a 10% (3/30) incidence (*p* = 0.469). Both control groups had a 6.7% (2/30) peritonitis rate, but the Propolis group had 0% peritonitis (*p* = 0.997). Survival analysis revealed no significant differences in ESI (*p* = 0.228) or peritonitis incidence times (*p* = 0.358). Although not statistically significant, Propolis completely prevented infection, while rates of 6.7%–10% were observed in controls, indicating clinical significance.

Duarte Silveira et al. (2022) investigated the anti‐inflammatory effects of a standardized Brazilian green Propolis extract (EPP‐AF) in 37 patients undergoing hemodialysis over an 8‐week intervention period (4 weeks of 200 mg/day EPP‐AF followed by 4 weeks' posttreatment). The intervention significantly reduced proinflammatory cytokines from baseline: IFN‐γ (median 12 pg/mL to 11 pg/mL, *p* = 0.005), IL‐13 (14 pg/mL to 10 pg/mL, *p* = 0.04), IL‐17 (15 pg/mL to 12 pg/mL, *p* = 0.039), IL‐1Ra (28 pg/mL to 25 pg/mL, *p* = 0.008), IL‐8 (556 pg/mL to 323 pg/mL, *p* = 0.009), and TNF‐α (155 pg/mL to 120 pg/mL, *p* < 0.001). These reductions were maintained after treatment for IFN‐γ (*p* = 0.003), IL‐10 (*p* = 0.03), IL‐12p70 (*p* < 0.001), and TNF‐α (*p* < 0.001), indicating lasting immunomodulatory effects. Heatmap analysis showed a pronounced baseline inflammatory state, especially in glomerulopathy patients, with notable attenuation following treatment. Hs‐CRP levels showed a trend toward improvement (11%–22% of patients reaching low‐risk levels, *p* = 0.3), although this was not statistically significant. No side effects were observed, and liver and pancreatic enzymes remained unchanged (*p* > 0.05) (Duarte Silveira et al. 2022).

Baptista et al. (2023) ran a randomized, double‐blind, placebo‐controlled trial to see how Brazilian Green Propolis extract (EPP‐AF) affects people on long‐term peritoneal dialysis. Nineteen patients with chronic kidney disease took part, with ten receiving 400 mg of the extract daily for two months and nine getting matching placebo capsules. The supplement led to a clear drop in plasma TNF‐α levels (*p* = 0.02) and tended to boost Nrf2 mRNA expression (*p* = 0.07), pointing to anti‐inflammatory and possible antioxidant action. However, no meaningful changes were found in IL‐6, CRP, or the lipid peroxidation marker malondialdehyde (MDA), all *p* > 0.05. Patients tolerated the treatment well and reported no serious side effects. Though limited by its small number of participants, the study hints that Propolis may reduce inflammation in dialysis patients, mainly by lowering TNF‐α.

Chermut et al. (2023) investigated the effect of standardized Brazilian green Propolis extract (EPP‐AF) in a double‐blinded, placebo‐controlled randomized clinical trial in 41 hemodialysis patients over a 2‐month intervention phase (400 mg/day). The Propolis subgroup (*n* = 21) evidenced a significant reduction in serum TNF‐α (*p* = 0.009) and trend toward decreased MIP‐1β (the macrophage inflammatory protein‐1β) (*p* = 0.07) from baseline, while none of the levels of IL‐2, IL‐6, IL‐7, IL‐8, IL‐10, IL‐17, or MCP‐1 were significantly changed (*p* > 0.05). The placebo group (*n* = 20) experienced no significant alteration in inflammatory markers. Biochemical markers, including albumin, phosphorus, and hemoglobin, remained unchanged in both groups (*p* > 0.05), testifying to the safety of the intervention with no adverse effect documented. The ability of Propolis to suppress inflammation among hemodialysis patients, particularly TNF‐α suppression, was revealed in the study, as supported by its documented NF‐κB inhibitory and antioxidant activities.

Fonseca, Alvarenga, et al. (2024) investigated the effects of standardized Brazilian green Propolis extract (EPP‐AF) in a randomized, double‐blind, placebo‐controlled study in 24 hemodialysis patients over a 2‐month intervention period (400 mg/day). The Propolis group (*n* = 9) showed a reduction in plasma concentration of oxLDL, from a median of 946 pg/mL (IQR 766–1212) to 824 pg/mL (IQR 700–1011) (*p* = 0.02), while the placebo group (*n* = 15) remained unchanged without statistical significance (1073 pg/mL [805–1331] to 825 pg/mL [695–1078], *p* = 0.13). The study demonstrated Propolis' ability to reverse oxidative stress among hemodialysis patients by reducing atherogenic oxLDL, a key promoter of cardiovascular risk among CKD patients. No side effects were reported, setting the safety of the intervention. It is, nonetheless, notable that these findings were submitted only in the form of a congress abstract at WCN24 (Buenos Aires, Argentina) and, upon request to the authors, no full‐text article was available for further methodological detail or further results. The abstract reported no conflicts of interest, but the limited availability of data means these preliminary results must be confirmed by peer‐reviewed publication.

Fonseca, Ribeiro, et al. (2024) conducted a randomized 8‐week trial with 400 mg/day of green Propolis supplementation in 42 hemodialysis patients (mean dialysis duration: 56–68 months). The study found no changes in plasma levels of uremic toxins after the intervention (indoxyl sulfate *p* = 0.24, p‐cresyl sulfate *p* = 0.77, indole‐3 acetic acid *p* = 0.09). Although microbial richness and evenness were slightly higher in the Propolis group (*n* = 6 subgroup analysis), there was no significant change in microbiota composition reflected by beta diversity metrics. Baseline correlations were observed between uremic toxins and markers of inflammation (IAA‐TNF‐α: *r* = 0.53, *p* = 0.01; IAA‐IL‐2: *r* = 0.66, *p* = 0.002; pCS‐IL‐7: *r* = 0.46, *p* = 0.04). Microbial analysis showed that Fusobacteria were positively correlated with IS (*p* < 0.05), whereas Firmicutes, Lentisphaerae, and Proteobacteria had negative correlations. Despite these correlations, the 8‐week treatment did not significantly change toxin levels or microbiota structure compared to placebo.

## 4 Discussion

This systematic review reported the latest evidence of the therapeutic benefits of Propolis supplementation in patients undergoing various forms of dialysis therapy. Overall findings from the studies included herein indicate that Propolis has beneficial effects on certain clinically relevant parameters in this patient group at high risk of adverse health events. While most of the studies incorporated within the analysis had positive results, particularly about inflammatory markers and oxidative variables of stress, mention might be made that there were investigations that failed to demonstrate statistically significant differences at certain of the endpoints measured. Of major clinical interest is the general safety profile observed in all of the included studies; no serious adverse effects having been reported regarding the administration of Propolis (Hu et al. 2023). However, isolated reports of adverse renal events, including acute renal failure described in previous literature (Rajoo et al. 2014), warrant caution when interpreting the safety profile of Propolis, particularly in patients with underlying kidney disease. Therefore, although current findings suggest that Propolis may be reasonably well tolerated, the available evidence remains insufficient to establish its safety conclusively.

The third most important bee product is Propolis, or “bee glue.” Bees produce it by mixing plant resins with wax and saliva, using it to defend their hives (Hu et al. 2023). It is identified by its color (green, red, brown) and melts between 65°C and 100°C (Papa et al. 2022). Propolis contains numerous bioactive compounds such as phenolic acids, flavonoids, and terpenes, which give it strong antioxidant activity. The polyphenol content varies greatly between samples, ranging from 143 to 324 mg gallic acid equivalents/g and 206–705 mg quercetin equivalents/g in ethanolic extracts (Papa et al. 2022). Its composition depends on geographic location, hive type, and season which makes establishing a universal dosage remain difficult (Braakhuis 2019; Laura Cornara et al. 2017). The most studied health benefits include antimicrobial effects, wound healing, and cardio protection (Papa et al. 2022; Rasul et al. 2013). One key component, CAPE, demonstrates powerful anti‐inflammatory activity by inhibiting COX enzymes (Kim et al. 2013; Wagh 2013), and modulating NF‐κB signaling (Arvouet‐Grand et al. 1993). CAPE reduces pro‐inflammatory cytokines (TNF‐α, IL‐8), increases anti‐inflammatory cytokines (IL‐10, IL‐4) (Doğanyiğit et al. 2020), and decreases inflammatory cell infiltration (Doğanyiğit et al. 2020; Silveira et al. 2021; Teles et al. 2015). Animal studies indicate a safe dose in humans of 70 mg/day (34), with low toxicity (LD 50 > 7.34 g/kg in mice) (Bazmandegan et al. 2017). It is nontoxic even at high doses (up to 20 g/kg) in rats (Mirzoeva and Calder 1996). Propolis also protects against oxidative stress and organ damage and improves renal function in sepsis models by reducing inflammation and macrophage infiltration (Moura et al. 2011; Orban et al. 2000; Zhao et al. 2014). Possible proteinuria reduction among CKD patients has been reported although this requires further investigations and in vivo studies (Abdel‐Latif et al. 2005). Generally well tolerated, with infrequent reports of allergic reactions and acute renal failure (Rajoo et al. 2014). The mechanism of kidney toxicity remains unclear, though rechallenge studies support an association (Rajoo et al. 2014). Propolis helps maintain endothelial health and modulates inflammation through various mechanisms, including COX inhibition, free radical scavenging, and cytokine regulation (Cornara et al. 2017). Overall, Propolis offers notable health benefits but needs more research to determine optimal dosing and identify rare adverse effects. An important challenge in interpreting the available evidence is the substantial variability in the chemical composition of Propolis. The concentration of biologically active constituents, including polyphenols and CAPE, differs considerably according to geographic origin, botanical source, harvesting season, and extraction methods (Cornara et al. 2017; Papa et al. 2022; Zulhendri et al. 2021). This variability creates a “black‐box” effect that limits reproducibility and comparability across clinical studies. Therefore, future randomized controlled trials should preferentially employ chemically standardized Propolis extracts with detailed characterization of their bioactive constituents to enable more reliable evaluation of efficacy and safety. Moreover, differences in the bioavailability of Propolis bioactive compounds, particularly among dialysis patients who may exhibit altered absorption, metabolism, and elimination, should also be considered when interpreting treatment responses and comparing findings across studies.

In this study, we assessed the effects of Propolis on various parameters in dialysis patients receiving different therapeutic regimens and/or medications.

With regard to the effect of Propolis on catheter exit‐site infection and peritonitis in patients undergoing peritoneal dialysis, the use of a 10% Propolis extract ointment applied every other day for 6 months resulted in catheter site infections in 10% of the placebo group and 6.7% of the control group, whereas no cases of infection were observed in the intervention group. Yet there were no significant differences in the incidence of catheter exit site infection and peritonitis among the three groups. This trial concluded that even though Propolis supplementation did not show any statistically significant changes in ESI or peritonitis rate, compared to the typical treatment with Mupirocin, which is a chemically‐derived drug and often leads to drug resistance, Propolis is plant‐based and does not produce drug resistance, so Propolis is recommended (Moghiseh et al. 2022).

Moreover, Duarte Silveira et al. (2022) reported a significant reduction in IFN‐γ, IL‐13, IL‐17, IL‐1Ra, IL‐8, and TNF‐α levels compared to baseline; the Hs‐CRP levels were reported as improved insignificantly after Propolis supplementation (200 mg/day) for 4 weeks of duration and a 1‐week washout period in between. A trend toward sustained reduction was observed even after discontinuation of EPP‐AF. No significant adverse effects were reported. This investigation was a prospective, open‐label, proof‐of‐concept, single‐center clinical study and did not include a placebo control group. Therefore, a stronger trial with blinded placebo‐controlled participants is recommended to better evaluate these results.

Baptista et al. (2023) also reported a significant reduction of TNF‐α plasma levels, and expression of Nrf2 showed a trend to increase after Propolis supplementation (400 mg/day) for 2 months of duration. However, no statistical change in MDA, IL‐6, and CRP plasma levels was observed. EPP‐AF Green Propolis extract supplementation appears as a potential strategy to mitigate inflammation, reducing TNF‐α plasma levels in CKD patients on PD.

Moreover, Fonseca, Alvarenga, et al. (2024) studied the effects of Propolis on Ox‐LDL plasma levels in patients undergoing hemodialysis. The patients in the intervention group received 400 mg/day concentrated and standardized dry EPP‐AF green Propolis extract capsules for 2 months. The intervention resulted in a decrease in Ox‐LDL plasma levels among the intervention group, but the difference between the placebo and intervention group was not statistically significant.

Fonseca, Ribeiro, et al. (2024) investigated the effects of Propolis supplementation on gut microbiota composition and uremic toxin profiles in patients undergoing hemodialysis. At baseline, they found a positive correlation between IAA and TNF‐α and IL‐2, as well as between pCS and IL‐7. Participants in the Propolis group received 400 mg/day of green Propolis extract for 8 weeks. The findings showed no significant changes in uremic toxin levels following the intervention. However, although not statistically significant, microbial evenness and observed richness increased after Propolis supplementation. Additionally, Fusobacteria abundance was positively associated with IS, whereas Firmicutes, Lentisphaerae, and Proteobacteria were negatively correlated with IS.

And finally, another study reported a significant reduction of the serum levels of TNF‐α and also a tendency of MIP‐1β levels under Propolis supplementation (400 mg/day) in 2 months of duration (Chermut et al. 2023).

### 4.1 Study Limitations and Need for Further Research

The current body of work, while heartening, is not uncomplicated by limitations that must be carefully considered in understanding these findings as well as planning future research. The most prominent aspect of methodological limitations is considerable heterogeneity in study designs, variability in dialysis modalities, diversity in patient populations and baseline characteristics (such as BMI ranges and renal disease stages), lack of standardization of Propolis preparations and dosing regimens, and a great extent of variability in treatment durations across studies. Additional confounding effects of ethnic differences, although examined directly in just one study, are also apt to influence treatment effects and the generalizability of findings. The lack of standardization in outcomes measured and analyses performed has made direct comparison of studies particularly challenging and has prevented useful meta‐analytic approaches to data synthesis in this systematic review. Furthermore, the included studies differed not only in dosage regimens and treatment duration but also in the route of administration, including topical and oral formulations. These methodological differences may influence the absorption, systemic exposure, and biological effects of Propolis, thereby reducing the overall comparability of study outcomes. Consequently, caution is warranted when interpreting pooled evidence across studies with substantially different intervention protocols. Taken together, these limitations emphasize the importance of conducting more standardized and methodologically robust clinical trials in this emerging area of research.

Future studies need to cover several priority areas to advance our understanding of the therapeutic potential of Propolis in dialysis patients. First, large‐scale multicenter randomized controlled trials involving chemically standardized Propolis extracts with quantified concentrations of major bioactive compounds (e.g., total polyphenols and CAPE), optimized dosage regimens, and treatment durations sufficient to allow adequate assessment are needed to establish definitive efficacy and safety profiles. Second, properly designed dose‐ranging investigations will be needed to establish optimum therapeutic doses for different clinical endpoints. Third, mechanistic research with advanced omics technologies can elucidate the molecular modes of action of Propolis and identify candidate biomarkers of response to treatment. Fourth, trials of long‐term outcomes are needed to determine whether the biochemical changes observed translate into clinically relevant benefits concerning reduced hospitalization rates, improved quality of life, or enhanced survival. Finally, detailed investigations of the modulation of the gut microbiome and its implications for systemic inflammation and uremic toxicity can provide new insights into additional mechanisms of action.

## 5 Conclusion

In conclusion, this systematic review combines evidence suggesting that Propolis supplementation may be associated with different benefits for dialysis patients, notably through its anti‐inflammatory and anti‐oxidant activity, although the current evidence remains limited and preliminary. Some studies reported favorable findings; however, methodological heterogeneity, small sample sizes, and the limited geographic diversity of the included studies reduce the certainty and generalizability of these observations. In addition, the available data are insufficient to draw firm conclusions regarding safety or risk–benefit balance. Appropriately designed, adequately powered randomized controlled trials involving standardized preparations of Propolis, appropriate outcome measures, and longer follow‐up periods are urgently needed before these findings can be translated into evidence‐based clinical practice. At present, Propolis should be considered an investigational adjunct rather than an established therapeutic option in dialysis care. Overall, the present systematic review supports Propolis as a promising adjunctive intervention for patients undergoing dialysis; however, the current evidence remains insufficient because of methodological heterogeneity and the lack of standardized Propolis formulations. Future well‐designed clinical trials using chemically characterized and standardized extracts are essential before definitive clinical recommendations can be made. While the available results appear encouraging, larger and more rigorous standardized RCTs are required to confirm efficacy, safety, and optimal dosing regimens.

## Author Contributions

**Mahshid Mardani:** conceptualization, methodology, data curation, formal analysis, resources, investigation, validation, writing – original draft. **Azizeh Farshbaf‐Khalili:** conceptualization, data curation, formal analysis, investigation, writing – review and editing. **Alireza Ostadrahimi:** data curation, resources, visualization, supervision, writing – review and editing.

## Funding

This article was financially supported by the Vice Chancellor for Research and Technology, Tabriz University of Medical Sciences (grant no: 76411).

## Consent

The authors have nothing to report.

## Conflicts of Interest

The authors declare no conflicts of interest.

## Supporting information


**Appendix S1:** Search strategy.

## Data Availability

The data presented in this study are available on request from the corresponding author.
