# Supplementary material for: Therapeutic Effects of Propolis Supplementation on the Inflammatory State, Oxidative Stress, and Uremic Toxins in dialysis Patients: A Systematic Review
Source: Food Sci Nutr. 2026 Sep 8;14(9):e72309. doi: 10.1002/fsn3.72309 (PMC13554138; doi:10.1002/fsn3.72309)
Supplement: Supplementary file 1 [file FSN3-14-e72309-s001.docx]

Supplementary Appendix 1: Search Strategy

PUBMED

(("Renal Dialysis"[Mesh]) OR ("hemodialysis"[Text Word] OR "blood dialysis"[Text Word] OR "dialysis center"[Text Word] OR "dialysis, blood"[Text Word] OR "extracorporeal blood cleansing"[Text Word] OR "extracorporeal dialysis"[Text Word] OR "haemodialysis"[Text Word] OR "haemodialysis center"[Text Word] OR "haemodialysis centre"[Text Word] OR "haemodialysis department"[Text Word] OR "haemodialysis unit"[Text Word] OR "haemodialysis units, hospital"[Text Word] OR "hemodialyse"[Text Word] OR "hemodialysis"[Text Word] OR "hemodialysis center"[Text Word] OR "hemodialysis department"[Text Word] OR "hemodialysis unit"[Text Word] OR "hemodialysis units, hospital"[Text Word] OR "hemorenodialysis"[Text Word] OR "hemotrialysate"[Text Word] OR "hospital haemodialysis units"[Text Word] OR "hospital hemodialysis units"[Text Word] OR "renal dialysis"[Text Word] OR "continuous ambulatory peritoneal dialysis"[Text Word] OR "capd"[Text Word] OR "ambulatory continuous peritoneal dialysis"[Text Word] OR "continuous ambulant peritoneal dialysis"[Text Word] OR "continuous ambulatory peritoneal dialysis"[Text Word] OR "peritoneal dialysis, continuous ambulatory"[Text Word] OR "peritoneal dialysis"[Text Word] OR "dialysis, peritoneal"[Text Word] OR "peritoneal dialysis"[Text Word] OR "peritoneum dialysis"[Text Word] OR "retina tear"[Text Word] OR "rpe tear"[Text Word] OR "rpe tears"[Text Word] OR "retina break"[Text Word] OR "retina hole"[Text Word] OR "retina macula tear"[Text Word] OR "retina perforation"[Text Word] OR "retina rupture"[Text Word] OR "retina tear"[Text Word] OR "retinal perforations"[Text Word] OR "retinal pigment epithelial tear"[Text Word] OR "retinal pigment epithelial tears"[Text Word] OR "retinal pigment epithelium tear"[Text Word] OR "retinal pigment epithelium tears"[Text Word] OR "retinal rupture"[Text Word] OR "retinal tear"[Text Word] OR "retinal tears"[Text Word] OR "ruptura retinae"[Text Word] OR "rupture, retina"[Text Word] OR "tear, retina"[Text Word] OR "extracorporeal dialyses"[Text Word] OR "dialysis"[Text Word] OR "acute dialysis"[Text Word] OR "coil dialysis"[Text Word] OR "dialysis"[Text Word] OR "flow dialysis"[Text Word])) AND (("Propolis"[Mesh]) OR ("propolis"[Text Word] OR "aagard"[Text Word] OR "bee cement"[Text Word] OR "bee glue"[Text Word] OR "bee resin"[Text Word] OR "hive dross"[Text Word] OR "propolis"[Text Word] OR "bee bread"[Text Word] OR "glue, bee"[Text Word]))

EMBASE

('hemodialysis'/exp OR 'blood dialysis':ti,ab,kw OR 'dialysis center':ti,ab,kw OR 'dialysis, blood':ti,ab,kw OR 'extracorporeal blood cleansing':ti,ab,kw OR 'extracorporeal dialysis':ti,ab,kw OR 'haemodialysis':ti,ab,kw OR 'haemodialysis center':ti,ab,kw OR 'haemodialysis centre':ti,ab,kw OR 'haemodialysis department':ti,ab,kw OR 'haemodialysis unit':ti,ab,kw OR 'haemodialysis units, hospital':ti,ab,kw OR 'hemodialyse':ti,ab,kw OR 'hemodialysis':ti,ab,kw OR 'hemodialysis center':ti,ab,kw OR 'hemodialysis department':ti,ab,kw OR 'hemodialysis unit':ti,ab,kw OR 'hemodialysis units, hospital':ti,ab,kw OR 'hemorenodialysis':ti,ab,kw OR 'hemotrialysate':ti,ab,kw OR 'hospital haemodialysis units':ti,ab,kw OR 'hospital hemodialysis units':ti,ab,kw OR 'renal dialysis':ti,ab,kw OR 'continuous ambulatory peritoneal dialysis'/exp OR 'capd':ti,ab,kw OR 'ambulatory continuous peritoneal dialysis':ti,ab,kw OR 'continuous ambulant peritoneal dialysis':ti,ab,kw OR 'continuous ambulatory peritoneal dialysis':ti,ab,kw OR 'peritoneal dialysis, continuous ambulatory':ti,ab,kw OR 'peritoneal dialysis'/exp OR 'dialysis, peritoneal':ti,ab,kw OR 'peritoneal dialysis':ti,ab,kw OR 'peritoneum dialysis':ti,ab,kw OR 'retina tear'/exp OR 'rpe tear':ti,ab,kw OR 'rpe tears':ti,ab,kw OR 'retina break':ti,ab,kw OR 'retina hole':ti,ab,kw OR 'retina macula tear':ti,ab,kw OR 'retina perforation':ti,ab,kw OR 'retina rupture':ti,ab,kw OR 'retina tear':ti,ab,kw OR 'retinal perforations':ti,ab,kw OR 'retinal pigment epithelial tear':ti,ab,kw OR 'retinal pigment epithelial tears':ti,ab,kw OR 'retinal pigment epithelium tear':ti,ab,kw OR 'retinal pigment epithelium tears':ti,ab,kw OR 'retinal rupture':ti,ab,kw OR 'retinal tear':ti,ab,kw OR 'retinal tears':ti,ab,kw OR 'ruptura retinae':ti,ab,kw OR 'rupture, retina':ti,ab,kw OR 'tear, retina':ti,ab,kw OR 'extracorporeal dialyses':ti,ab OR 'dialysis'/exp OR 'acute dialysis':ti,ab,kw OR 'coil dialysis':ti,ab,kw OR 'dialysis':ti,ab,kw OR 'flow dialysis':ti,ab,kw) AND ('propolis'/exp OR 'aagard':ti,ab,kw OR 'bee cement':ti,ab,kw OR 'bee glue':ti,ab,kw OR 'bee resin':ti,ab,kw OR 'hive dross':ti,ab,kw OR 'propolis':ti,ab,kw OR 'bee bread'/exp OR 'glue, bee':ti,ab)

WOS (web of science)

"hemodialysis" OR "blood dialysis" OR "dialysis center" OR "dialysis, blood" OR "extracorporeal blood cleansing" OR "extracorporeal dialysis" OR "haemodialysis" OR "haemodialysis center" OR "haemodialysis centre" OR "haemodialysis department" OR "haemodialysis unit" OR "haemodialysis units, hospital" OR "hemodialysed" OR "hemodialysis" OR "hemodialysis center" OR "hemodialysis department" OR "hemodialysis unit" OR "hemodialysis units, hospital" OR "hemorenodialysis" OR "hemodialysate" OR "hospital haemodialysis units" OR "hospital hemodialysis units" OR "renal dialysis" OR "continuous ambulatory peritoneal dialysis" OR "capd" OR "ambulatory continuous peritoneal dialysis" OR "continuous ambulant peritoneal dialysis" OR "continuous ambulatory peritoneal dialysis" OR "peritoneal dialysis continuous ambulatory" OR "peritoneal dialysis" OR "dialysis, peritoneal" OR "peritoneal dialysis" OR "peritoneum dialysis" OR "retina tear" OR "rpe tear" OR "rpe tears" OR "retina break" OR "retina hole" OR "retina macula tear" OR "retina perforation" OR "retina rupture" OR "retina tear" OR "retinal perforations" OR "retinal pigment epithelial tear" OR "retinal pigment epithelial tears" OR "retinal pigment epithelium tear" OR "retinal pigment epithelium tears" OR "retinal rupture" OR "retinal tear" OR "retinal tears" OR "ruptura retinae" OR "rupture retina" OR "tear retina" OR "extracorporeal dialyses" OR "dialysis" OR "acute dialysis" OR "coil dialysis" OR "dialysis" OR "flow dialysis" (Topic) and "propolis" OR "asgard" OR "bee cement" OR "bee glue" OR "bee resin" OR "hive dross" OR "propolis" OR "bee bread" OR "glue, bee" (Topic) | 13

SCOPUS

( TITLE-ABS-KEY ( "hemodialysis" OR "blood dialysis" OR "dialysis center" OR "dialysis, blood" OR "extracorporeal blood cleansing" OR "extracorporeal dialysis" OR "haemodialysis" OR "haemodialysis center" OR "haemodialysis centre" OR "haemodialysis department" OR "haemodialysis unit" OR "haemodialysis units, hospital" OR "hemodialyse" OR "hemodialysis" OR "hemodialysis center" OR "hemodialysis department" OR "hemodialysis unit" OR "hemodialysis units, hospital" OR "hemorenodialysis" OR "hemotrialysate" OR "hospital haemodialysis units" OR "hospital hemodialysis units" OR "renal dialysis" OR "continuous ambulatory peritoneal dialysis" OR "capd" OR "ambulatory continuous peritoneal dialysis" OR "continuous ambulant peritoneal dialysis" OR "continuous ambulatory peritoneal dialysis" OR "peritoneal dialysis, continuous ambulatory" OR "peritoneal dialysis" OR "dialysis, peritoneal" OR "peritoneal dialysis" OR "peritoneum dialysis" OR "retina tear" OR "rpe tear" OR "rpe tears" OR "retina break" OR "retina hole" OR "retina macula tear" OR "retina perforation" OR "retina rupture" OR "retina tear" OR "retinal perforations" OR "retinal pigment epithelial tear" OR "retinal pigment epithelial tears" OR "retinal pigment epithelium tear" OR "retinal pigment epithelium tears" OR "retinal rupture" OR "retinal tear" OR "retinal tears" OR "ruptura retinae" OR "rupture, retina" OR "tear, retina" OR "extracorporeal dialyses" OR "dialysis" OR "acute dialysis" OR "coil dialysis" OR "dialysis" OR "flow dialysis" ) AND TITLE-ABS-KEY ( "propolis" OR "aagard" OR "bee cement" OR "bee glue" OR "bee resin" OR "hive dross" OR "propolis" OR "bee bread" OR "glue, bee" ) )
